# Pituitary thyrotoxicosis presenting as abnormal thyroid function testing during pregnancy: a case report

**DOI:** 10.1186/s13256-021-02734-4

**Published:** 2021-04-04

**Authors:** David George Jackson, John Parker, Thomas Cummings

**Affiliations:** 1 Wilmington Health Endocrinology, 2421 Silver Stream Lane, Wilmington, NC 28401 USA; 2 Department of Pathology, Duke University, Durham, NC USA

**Keywords:** Pituitary, Macroadenoma, Thyrotoxicosis, Pregnancy

## Abstract

**Background:**

Central hyperthyroidism is a rare form of hyperthyroidism caused by thyrotrope pituitary adenomas. It is characterized by elevated thyroid-stimulating hormone alongside high thyroxine and triiodothyronine. Goiter is the most common symptom of central hyperthyroidism. Surgical resection as well as somatostatin analog therapy typically achieve resolution of hyperthyroid symptoms and restoration of a euthyroid state.

**Case presentation:**

We report the case of a 30-year-old primigravida Caucasian/White female who presented with abnormal thyroid function testing results and multinodular goiter during pregnancy. Postpartum, she was found to have multinodular goiter on physical examination as well as persistent elevated thyroid-stimulating hormone with elevated free thyroxine and free triiodothyronine. Magnetic resonance imaging disclosed a large pituitary macroadenoma, and she subsequently underwent resection of the mass. She achieved a sustained euthyroid state postoperatively.

**Conclusions:**

This case shows how central hyperthyroidism can present without the more apparent symptoms of thyrotoxicosis and that successful resolution of central hyperthyroidism may be achieved postoperatively.

## Background

Hyperthyroidism is the most common cause of thyrotoxicosis. It is characterized by excessive activity of the thyroid gland and can manifest with symptoms such as tachycardia, palpitations, unintentional weight loss, diaphoresis, anxiety, and exophthalmos, especially in the case of Graves’ disease. The majority of cases of thyrotoxicosis are due to autoimmune phenomena (particularly Graves’ disease) as well as inflammatory phenomena (such as postviral subacute thyroiditis). Rare cases of hyperthyroidism are due to pituitary thyrotrope adenomas (a phenomenon called central hyperthyroidism), with an approximate prevalence of one per million for these adenomas [1]. Pituitary thyrotrope adenomas are almost entirely benign and secrete thyroid-stimulating hormone (TSH) to stimulate the thyroid gland; thus, characteristic laboratory findings of central hyperthyroidism are elevated TSH, T4, and T3 levels. TSH-related compounds (especially the alpha subunit of glycoprotein hormones) also tend to be elevated in central hyperthyroidism [2]. The majority of thyrotrope pituitary adenomas are macroadenomas [3]. Thyrotrope macroadenomas frequently secrete more than one type of hormone (including growth hormone and prolactin), with 10 out of 21 patients in one study having ten plurihormonal adenomas and another study finding 14 of 90 thyrotrope adenoma patients with acromegaly [3, 4]. Typical central hyperthyroid symptoms are less severe than the common autoimmune hyperthyroid symptoms and are usually limited to goiter and palpitations with rarer instances of cardiac arrhythmias [1, 3]. Hyperthyroidism affects approximately 0.1–0.4% of all pregnancies [5]. During pregnancy, hCG (human chorionic gonadotropin) stimulates the TSH receptor and increased levels of thyroxine-binding globulin are present [6]. Increased stimulation of the TSH receptor may exacerbate a hyperthyroid state [7]. Hyperthyroidism can have deleterious effects on pregnancy, including maternal hypertension, low birth weight, preeclampsia, heart failure, and even spontaneous abortion [8]. Diagnosis of central hyperthyroidism is typically accomplished by measuring elevated TSH in the context of high T4 and T3 when compared with the reference range, and thyrotrope macroadenomas are usually found to be hypoenhancing macroadenomas when imaged with magnetic resonance imaging (MRI) [4, 9]. Thyrotropin-releasing hormone stimulation testing and T3 suppression testing are used to confirm the diagnosis of central hyperthyroidism [1]. Although diagnosis of TSH-secreting pituitary macroadenoma appears straightforward, there has been considerable diagnostic delay in many cases, with one study finding a mean diagnostic delay of approximately 6 years [10].

Surgical removal of the thyrotrope pituitary adenoma is the first-line treatment of thyrotrope pituitary macroadenomas [1]. Antithyroid drugs such as methimazole or propylthiouracil are used to achieve euthyroidism before resection of the adenoma. Surgical success rates are higher if the diagnosis is made earlier, and the effectiveness of the surgery may be diminished if the adenoma extends into the extrasellar and parasellar regions [1, 3]. If this treatment fails, somatostatin analog therapy is typically used. This treatment both decreases circulating TSH levels and shrinks goiters. For patients who did not achieve complete resolution of central hyperthyroidism after pituitary resection, postoperative radiation or somatostatin analog therapy typically yielded favorable outcomes. No exact criteria currently exist to characterize successful remission of a thyrotrope adenoma, so success rates are usually determined by resolution of hyperthyroid symptoms and normalization of TSH, T4, and T3 [1, 4]. Follow-up is typically done through two to three visits during the first postoperative year and once-yearly visits thereafter, and recurrence of the TSH-secreting adenoma does not typically occur.

## Case presentation

**Fig. 1:**
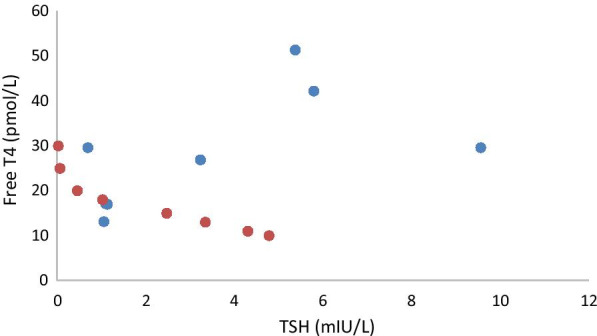
Aberrant relationship between free T4 and thyroid-stimulating hormone postpartum in our patient (blue) *versus* estimates of normal relationship by formulas from Hadlow *et al.* [9] (red). Free T4 and TSH are given in pmol/L and mIU/L, respectively, to be able to calculate a normal curve based on previously derived formulas [9]

**Fig. 2:**
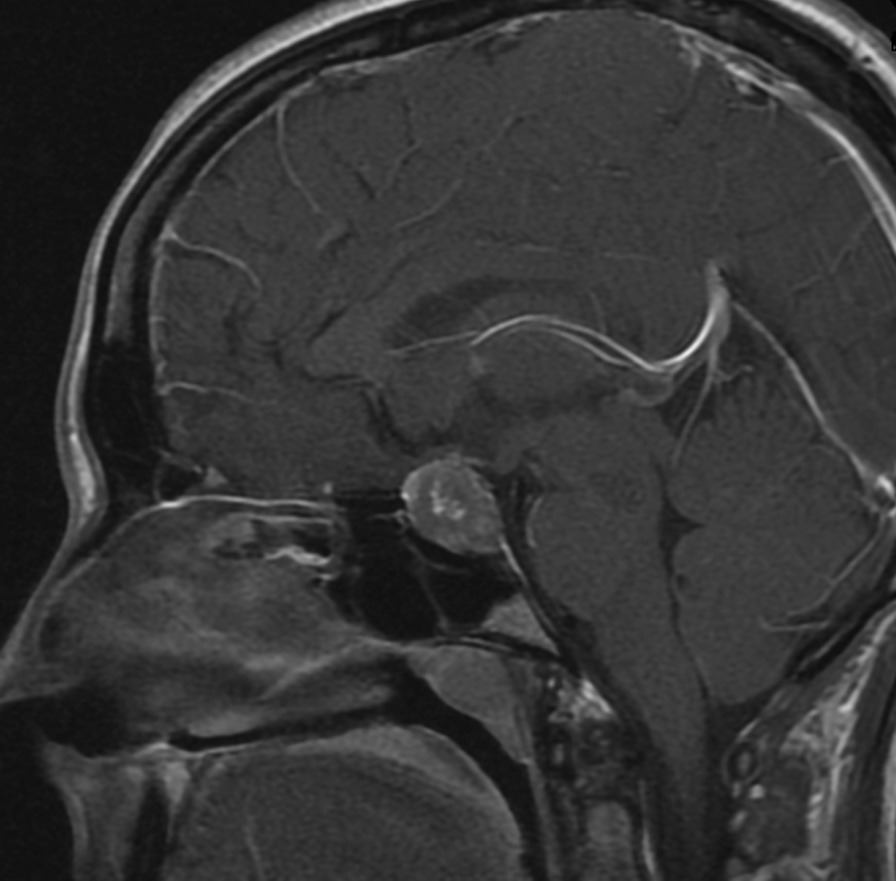
Magnetic resonance imaging showing pituitary macroadenoma with suprasellar extension and optic chiasm compression

**Fig. 3:**
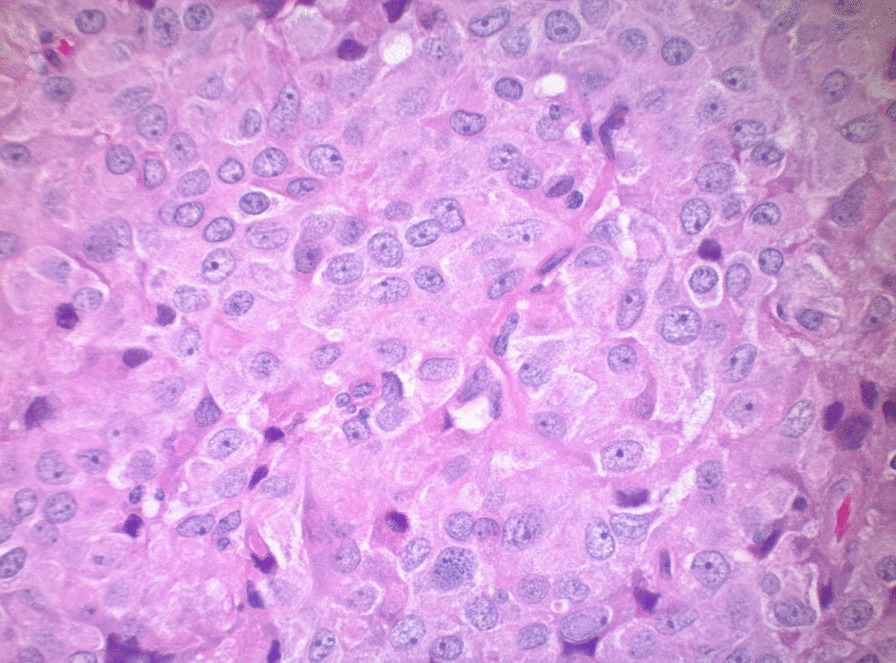
Pituitary adenoma characterized by a solid proliferation of cells with monomorphic nuclei and eosinophilic cytoplasm, hematoxylin and eosin ×40

**Fig. 4:**
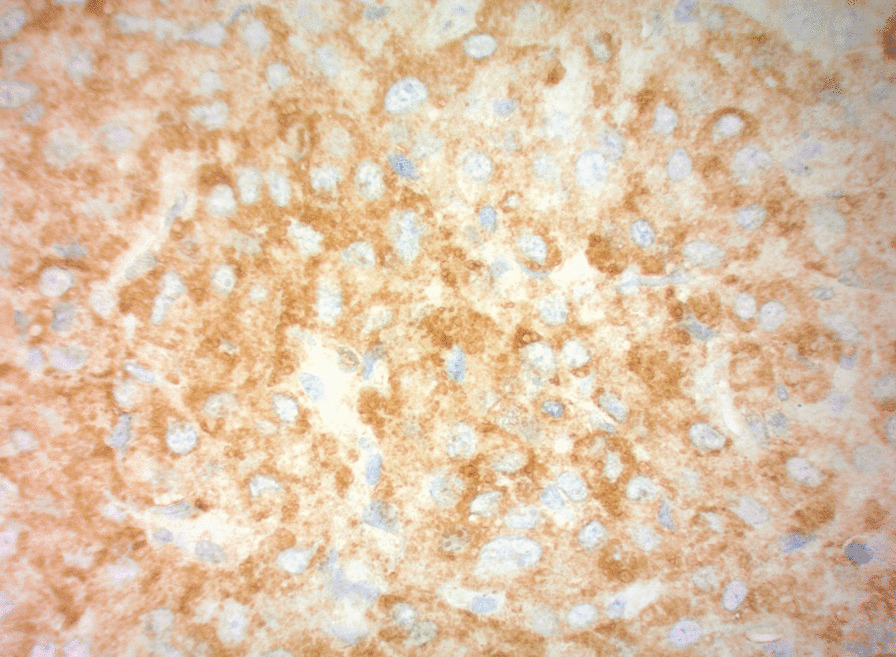
Strong positive immunohistochemical stain for thyroid-stimulating horm in neoplastic cells, ×40

We report on a 30-year-old gravida 1, para 0 (G1P0) Caucasian/White female with no past history of known thyroid abnormalities or cervical compressive symptomatology who was found to have abnormal thyroid function testing results and multinodular goiter during her second trimester of pregnancy. Her past medical history is only significant for unspecified anxiety prior to her most recent pregnancy. She has been on no new medications and has no known drug allergies. Her family history is significant for a mother with hypothyroidism and an aunt with thyrotoxicosis. Social history is significant for a three cigarettes/day smoking history and past infrequent methamphetamine use. Physical examination demonstrated a soft, multinodular goiter without any additional physical signs of thyrotoxicosis such as proptosis, lid lag, tremor, tachycardia, or acropachy. Body mass index at presentation was 21.47, and blood pressure was 133/82 mmHg. Thyroid testing revealed elevated TSH of 5.89 mIU/L with elevated total T4 of 24.6 nmol/L. Follow-up confirmed persistent elevation of TSH with elevation in total T4. She delivered a healthy, normal-weight male at 40 weeks gestation without complications. Postpartum, she was identified as having persistent abnormalities in thyroid hormone function testing and her TSH remained non-suppressed with unequivocally elevated T4 and T3 (Table 1, Fig. 1). Thyroid uptake by I-123 was elevated at 43% (normal range 7–33% at 24 h). Evaluation for the rare possibility of pituitary thyrotoxicosis disclosed pituitary macroadenoma. Pituitary MRI (Fig. 2) demonstrated a 17 × 17 × 20 mm macroadenoma of the pituitary with suprasellar extension and optic chiasm compression. Methimazole was commenced at 16 weeks postpartum to render euthyroid for surgery, and improvements in free T3 and free T4 were observed (Table 1, Fig. 1). Methimazole therapy was discontinued immediately preoperatively. She underwent pituitary resection at 32 weeks postpartum, and the surgical specimen demonstrated findings consistent with thyrotrope adenoma (Figs. 3, 4). Hematoxylin- and eosin-stained slides show a pituitary adenoma characterized by a diffuse and solid proliferation of generally monomorphic cells with round nuclei and eosinophilic cytoplasm. A few mitoses are present. There is no fibrosis, calcifications, or necrosis. By immunohistochemistry, the tumor cells are immunopositive for synaptophysin, chromogranin, and thyroid-stimulating hormone. Prolactin, human growth hormone, ACTH, LH, FSH, and beta-chorionic gonadotropin are negative in the tumor cells. She has done very well following her surgery and denied visual changes and headaches. She has continued nursing. She has maintained euthyroidism postoperatively and has had no additional pituitary hormone deficits.

**Table 1:** Numerical values of thyroid-stimulating hormone, total T4, free T3, and free T4 in our patient over time

Date	TSH (ref 0.55–4.78 mIU/L)	Total T4 (ref 57.92–136.42 nmol/L)	Free T3 (ref 3.07–6.76 pmol/L)	Free T4 (ref 7.85–22.65 pmol/L)	IGF-I (ref 73–243 μg/L)	Cortisol (ref 85.28–618.24 nmol/L)	FSH (ref 3.4–33.4 IU/L)	LH (ref 8.7–76.3 IU/L)	ACTH (ref 1.58–13.93 pmol/L)	Prl (ref 0.12–1.27 nmol/L)
19 weeks	5.890	316.60								
24 weeks	4.40	236.81								
26 weeks	1.45	257.40	6.41	21.62						
31 weeks	1.38	265.12								
38 weeks	2.26		7.24							
16 weeks PP	5.38		18.90	51.35						
19 weeks PP	5.8		18.96	42.21	269	270.48	5.47	7.29	7.46	
21 weeks PP	3.24			26.90						
25 weeks PP	9.57			29.60						
29 weeks PP	0.70		10.20	29.60		621.28				
32 weeks PP	1.14		5.53	16.99		326.51			1.94	
37 weeks PP	1.06		4.06	13.13		508.67	12.05	17.68		0.36
44 weeks PP	1.1		4.82	17.12	239		8.03	15.15		0.63

Data listed under red dates are lab values during pregnancy; data listed under blue dates are postpartum lab values. Methimazole was taken from postpartum weeks 16 through 29. PP designates postpartum weeks

## Discussion and conclusions

This case shows the subtlety and variation in presentation of a thyrotrope pituitary adenoma. Our patient presented with no obvious thyrotoxic symptoms (apart from a possible past history of anxiety, which is not specific to hyperthyroidism) and was only found to have central hyperthyroidism on the basis of physical examination findings and laboratory data. Our patient’s findings are somewhat comparable to trends found in a Japanese study of 90 patients with central hyperthyroidism since 85% of those patients presented with goiter as a main symptom [3]. Unlike our patient, 55% of the patients in the Japanese study group presented with palpitations or tachycardia as main symptoms as well. Also, our patient had no other symptoms consistent with excessive secretion of a second stimulatory hormone such as growth hormone or prolactin, while other patients have presented with acromegaly or symptoms of hyperprolactinemia that occur simultaneously with hyperthyroid symptoms [3, 4]. During pregnancy, the increased levels of hCG have a weak stimulatory effect on the TSH receptor and thus may contribute to hyperthyroidism, although TSH suppression is a hallmark [5, 6]. Because of lack of symptoms, our patient was only found to have hyperthyroidism through laboratory tests. Though total T4 was elevated during pregnancy, this is commonly a consequence of elevated thyroxine-binding globulin during pregnancy. Her albumin was mildly lowered at 3.3 g/dL, which is appropriate for pregnancy. Given her lack of symptoms, it is unlikely that further investigation into the possibility of pituitary adenoma would have occurred during pregnancy. Furthermore, because ongoing fetal assessment was normal, treatment was withheld in favor of clinical monitoring. The course of events would have been altered if the hyperthyroidism had been so severe that it had adversely impacted the pregnancy. Though hyperthyroidism can result in both preeclampsia and stillbirth, well controlled or asymptomatic hyperthyroidism is very unlikely to jeopardize a pregnancy [8].

Our case is also a good demonstration of successful medical and surgical management of a TSH-secreting pituitary adenoma. We prescribed our patient methimazole to assure normalization of thyroid hormone levels prior to resection of her adenoma. Some current evidence exists to support the use of methimazole in a preoperative setting, but no strong recommendations exist for using this therapy to attain preoperative euthyroidism [1]. Postoperatively, she maintained euthyroidism and demonstrated no recurrence of hyperthyroidism, so methimazole was discontinued. Her results are consistent with other treatment modalities; a study of 43 French and Belgian patients from 1976 to 2001 found that usage of somatostatin analogs typically reduced TSH levels by over 50% and that 58.3% of patients who underwent pituitary resection had a favorable outcome 1 year postsurgery [2].

In summary, thyrotrope adenomas are exceedingly rare secretory pituitary adenomas that cause central hyperthyroidism and can be managed with pituitary resection. Our patient shows typical findings of central hyperthyroidism as well as successful surgical management of the adenoma.

## Data Availability

The datasets used and/or analyzed during the current study are available from the corresponding author on reasonable request.

## Abbreviations

TSH: Thyroid-stimulating hormone
IGF-1: Insulin-like growth factor 1
FSH: Follicle-stimulating hormone
LH: Luteinizing hormone
ACTH: Adrenocorticotropic hormone
Prl: Prolactin
